# Kinematic synergies of hand grasps: a comprehensive study on a large publicly available dataset

**DOI:** 10.1186/s12984-019-0536-6

**Published:** 2019-05-28

**Authors:** Néstor J. Jarque-Bou, Alessandro Scano, Manfredo Atzori, Henning Müller

**Affiliations:** 1 Department of Mechanical Engineering and Construction, Universitat Jaume I, Castellón de la Plana, Spain; 2 Institute of Intelligent Industrial Systems and Technologies for Advanced Manufacturing (STIIMA), National Research Council of Italy (CNR), Milan, Italy; 3 Institute of Intelligent Industrial Systems and Technologies for Advanced Manufacturing (STIIMA), National Research Council of Italy (CNR), Lecco, Italy; 4 Information Systems Institute, University of Applied Sciences Western Switzerland (HES-SO), Sierre, Switzerland; 5 Medical Informatics, University of Geneva, Rue Gabrielle-Perret-Gentil 4, 1205 Geneva, Switzerland

**Keywords:** Cluster analysis, Cyberglove, Hand synergies, Kinematics, Myoelectric prostheses, Rehabilitation, Principal component analysis

## Abstract

**Background:**

Hand grasp patterns require complex coordination**.** The reduction of the kinematic dimensionality is a key process to study the patterns underlying hand usage and grasping. It allows to define metrics for motor assessment and rehabilitation, to develop assistive devices and prosthesis control methods. Several studies were presented in this field but most of them targeted a limited number of subjects, they focused on postures rather than entire grasping movements and they did not perform separate analysis for the tasks and subjects, which can limit the impact on rehabilitation and assistive applications. This paper provides a comprehensive mapping of synergies from hand grasps targeting activities of daily living. It clarifies several current limits of the field and fosters the development of applications in rehabilitation and assistive robotics.

**Methods:**

In this work, hand kinematic data of 77 subjects, performing up to 20 hand grasps, were acquired with a data glove (a 22-sensor CyberGlove II data glove) and analyzed. Principal Component Analysis (PCA) and hierarchical cluster analysis were used to extract and group kinematic synergies that summarize the coordination patterns available for hand grasps.

**Results:**

Twelve synergies were found to account for > 80% of the overall variation. The first three synergies accounted for more than 50% of the total amount of variance and consisted of: the flexion and adduction of the Metacarpophalangeal joint (MCP) of fingers 3 to 5 (synergy #1), palmar arching and flexion of the wrist (synergy #2) and opposition of the thumb (synergy #3). Further synergies refine movements and have higher variability among subjects.

**Conclusion:**

Kinematic synergies are extracted from a large number of subjects (77) and grasps related to activities of daily living (20). The number of motor modules required to perform the motor tasks is higher than what previously described. Twelve synergies are responsible for most of the variation in hand grasping. The first three are used as primary synergies, while the remaining ones target finer movements (e.g. independence of thumb and index finger). The results generalize the description of hand kinematics, better clarifying several limits of the field and fostering the development of applications in rehabilitation and assistive robotics.

## Background

The use of the hand to grasp and manipulate objects involves the coordination of a multitude of degrees of freedom (DoF) and the exploitation of redundancy both at kinematic and muscular level. As a consequence, the reduction of the dimensionality is a key process to understand hand usage patterns.

The hypothesis that motor modules are the basis of motor control at the neural level [1] (acting as reference motor primitives able to simplify the problem of motor control [2]) is often accepted in literature, even if it not all experimental data support it [3]. Dimensionality reduction for DoF of the hand (hand kinematic synergy extraction) is applied widely in research, for instance to study human grasps [4–12], hand prosthesis control [13–16], gesture recognition [17] and generally for rehabilitation [18, 19]. Many methods allow to achieve dimensionality reduction. Principal Component Analysis (PCA) is a well-known statistical procedure that uses an orthogonal transformation to remap a set of correlated variables into a smaller set of linearly uncorrelated variables called principal components (PCs): the highest variance is found in the first coordinate (first PC), the second highest variance in the second coordinate, etc. Each PC is therefore a vector including the content of each original variable.

Several scientific articles applied dimensionality reduction of hand kinematics to study grasping postures, as reported in Table 1 (that summarizes the main features of such work) and Table 2 (that summarizes the results in terms of extracted synergies). A study on hand grasps by Santello et al. [4] recorded 15 joint angles in five subjects while performing imaginary grasps. They found that the two main synergies accounted for more than 80% of the overall variation. The study also remarked that the remaining variation was due to motor control modules needed for fine tuning. Mason et al. [9] studied five types of reach-to-grasp movements on five subjects, showing that the first synergy accounted for more than 97% of the total variance. Liu et al. [10] studied postural grasps in ten subjects that were asked to grasp six objects (spheres, cylinders and prisms) in different relative positions between the human hand and objects, concluding that a reduced number of modules are needed to reproduce the original movement. Their results showed that the information transmitted by the PCs was lower than what was described in previous articles. The authors suggested that changes in the relative position between the human hand and the grasped objects can have an influence on grasp postures and that synergies are task-dependent [12]. Jarrassé et al. [11] investigated 15 DoF in ten subjects that were asked to “grasp-give-receive” nine objects. Four postural synergies were found: the first and second PCs accounted for approximately 90% of the data variation, although pattern refinement can be achieved by adding further PCs. In Patel et al. [8], ten subjects performed 25 grasps but only considering 10 DoF hand joints. While the first synergies account for more than half of the total variation, the remaining variation is distributed across many synergies, indicating that a large set of motor modules is needed to reconstruct the original kinematics. In Thakur et al. [6], eight subjects were asked to perform an unconstrained haptic exploration of 50 objects, so an uncontrolled setup. The objects were only explored, few grasping movements were made and not the entire movement (including the reach and release phases) was considered. Seven synergies encompassed over 90% of the total variance in the hand-grasps and motions, that were similar across subjects and across manipulations of different objects. In Todorov et al. [12], six right-handed subjects participated in an experiment including seven tasks. The results showed that the synergies differ substantially across subjects and tasks.

**Table 1:** Summary of selected previous studies on kinematic hand grasp synergies

Study	Subjects	Description of the task	Summary of the results
Santello et al. [4]	5	Grasp and use of 57 imagined objects	Two main synergies accounted for more than 80% of the overall variation.
Mason et al. [9]	5	5 reach-to-grasp movements with similar objects	First synergy accounted for more than 97% of the total variance.
Liu et al. [10]	10	Grasp six objects changing relative position of hand-objects	The first six PCs accounted for more than 80% of the total variance.
Jarrassé et al. [11]	10	Grasp postures of nine objects	Four postural synergies were found: the first and second PCs accounted for approximately 90% of the total variance.
Patel et al. [8]	10	Twenty-five grasping movements	Only 10 DoF were considered. First synergy accounted for approximately 54% of the variance.
Thakur et al. [6]	8	Haptic exploration over 50 objects Grasping postures with few objects	Seven synergies were extracted for the explanation of over 90% of the total variance.
Todorov et al. [12]	6	Seven tasks	Between two and four synergies (depending on the task) accounted for 85–95% of the overall variance.

**Table 2:** Number of kinematic synergies and variance explained by the three first synergies obtained in previous studies

Study	Total number of synergies	Variance explained by the three first synergies
Santello et al. [4]	14	∼90% of the variance
Mason et al. [9]	10	∼99.6% of the variance
Liu et al. [10]	15	∼65% of the variance
Jarrassé et al. [11]	4	∼93% of the variance
Patel et al. [8]	10	∼75% of the variance
Thakur et al. [6]	7	∼80% of the variance

As a consequence, there are several open points in the literature regarding hand grasp synergies that can be investigated in more detail. First, the most advanced studies involve a small number of subjects (the maximum number of subjects considered in a study is ten), limiting the possibility to generalize the results to bigger populations. Second, most of the studies map a small number of grasps compared to the number required for activities of daily living [20–23], thus affecting the possibility to use the results for applications targeting rehabilitation or assistive robotics. The only study including a comparable number of grasps (25 grasps), analyzes only 10 DoF, which is low in comparison to the DoF of the hand; the study included only 10 subjects [8]. Third, most studies analyze postural synergies and do not consider the whole movement, despite reach and release phases being a part of the coordination of daily life activity that is intimately related to hand grasp. Fourth, most studies do not specify if calibration procedures were used before analyzing the kinematic data, which is fundamental considering that the nonlinearities and imperfect kinematic representations are normally expected by measurement systems such as data gloves [24, 25]. Last, kinematic synergies are commonly extracted without distinguishing between tasks and subjects, although it is known that kinematic synergies are task and subject-dependent [12].

In order to contribute to clarifying the mentioned limits of current scientific literature, the aim of this study is to extract representative hand kinematic synergies from a publicly available database (NinaPro) [26]. The database includes a large number of subjects performing hand movements, much larger than previous studies. The correspondence between the considered hand movements and activities of daily living [23] also fosters the application of the results to improve rehabilitation and assistive robotics.

## Methods

### Acquisition setup

**Fig. 1:**
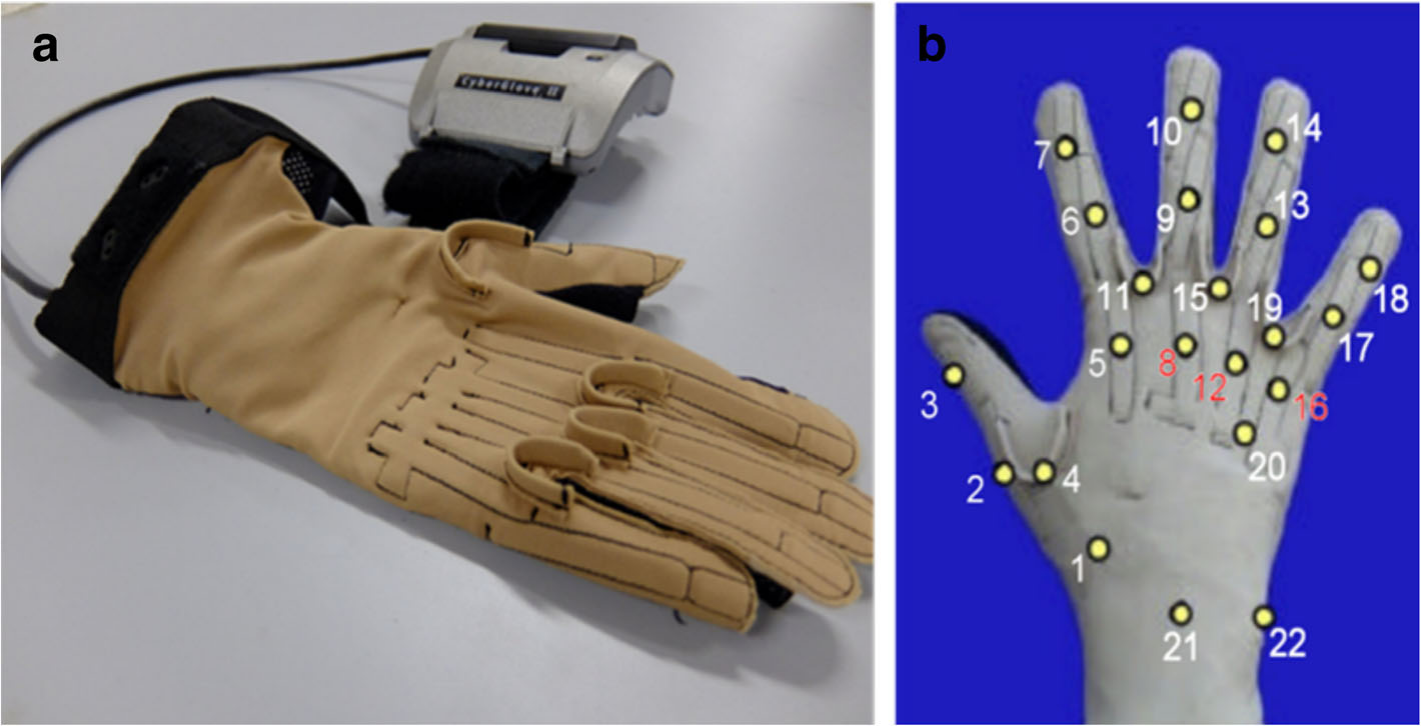
**a** Cyberglove II device. **b** The number and corresponding position of each Cyberglove sensor

The acquisition setup includes several sensors, designed to record hand kinematics, dynamics and the corresponding muscular activity. The sensors were connected to a laptop for data acquisition. Hand kinematics was measured using a 22-sensor CyberGlove II data glove (CyberGlove Systems LLC, www.cyberglovesystems.com). The CyberGlove is a motion capture data glove, instrumented with joint-angle measurements. It uses proprietary resistive bend-sensing technology to transform hand and finger motions into real-time digital joint-angle data, after an accurate calibration. Data from the CyberGlove were transmitted over a Bluetooth-tunneled serial port at slightly lower than 25 Hz. Each data sample provided was associated to an accurate timestamp using Windows performance counters. The number and corresponding position of each Cyberglove sensor is shown in Fig. 1. The acquisition setup is described in detail in [27, 28].

### Subjects

The data used in this experiment are from the publicly available NinaPro database. The complete dataset includes over 130 subjects performing up to 53 movements (the number of movements varies slightly between the data sets) while several sensors record the hand movements (e.g. sEMG, force sensors, data gloves, accelerometers, IMUs, eye trackers, …). This paper analyses the calibrated kinematic data of 77 subjects (NinaPro DB1, DB2, and DB5), performing 20 hand grasps. The three datasets were selected in order to have homogeneous acquisition setup and protocol. The subjects include 56 males, 21 females; 69 right handed, 8 left handed; average age 28.8 years with a standard deviation of 3.96 years (data summarized in Table 3). The calibrated kinematic data will be released as a separate dataset both on NinaPro^1^ and on Zenodo.^2^

**Table 3:** Description of the subjects

	Kinematic Ninapro
Available subjects	77
Considered subjects	77
Males	56
Females	21
Right-handed	69
Left-handed	8
Avg. Age (years)	28.8 ± 3.96
Avg. Height (cm)	172.6 ± 9.48
Avg. Weight (kg)	69.5 ± 11.97
Avg. BMI (Kg/m2)	23.06 ± 3.18

### Acquisition protocol

**Fig. 2:**
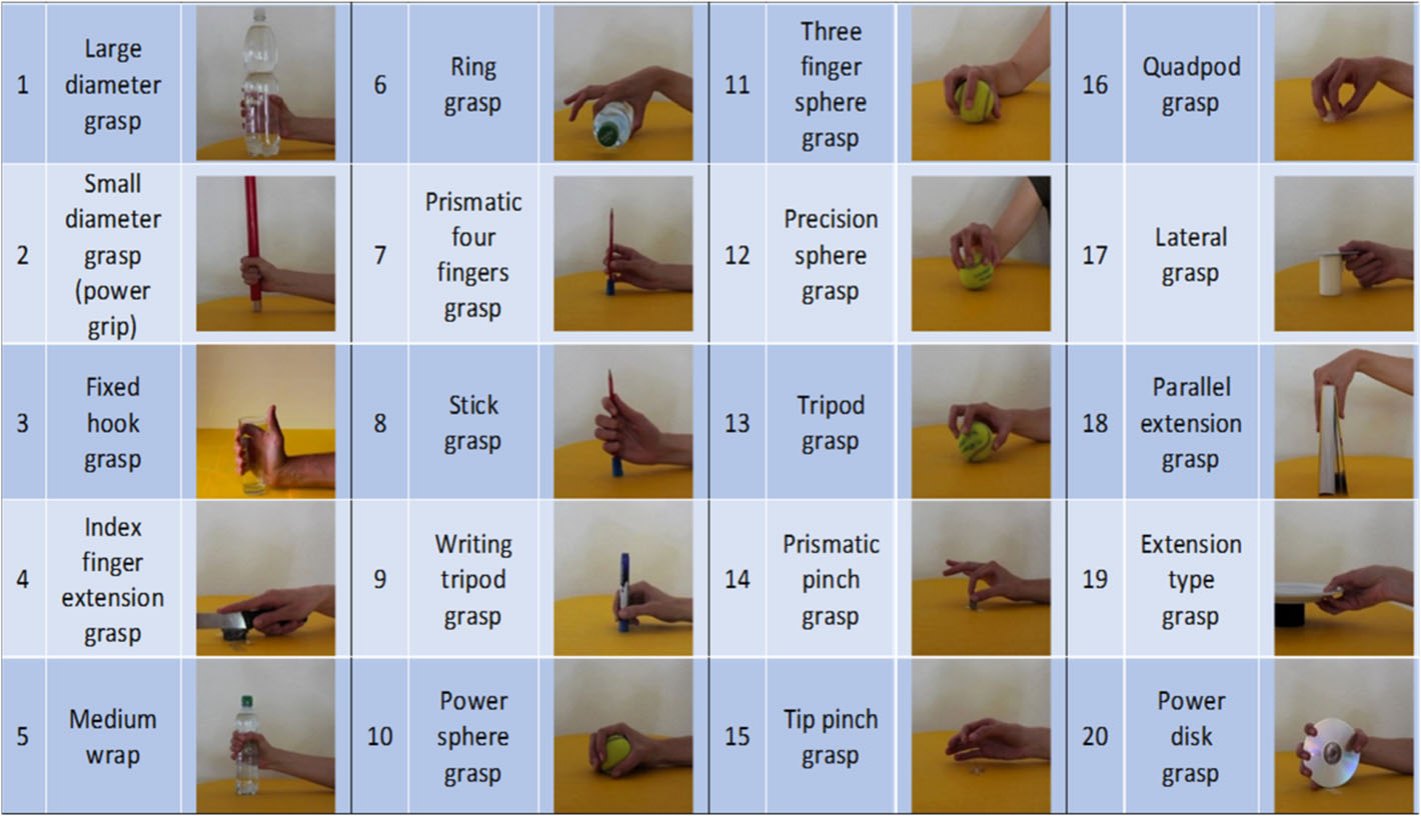
The 20 grasps considered in the study. They provide a comprehensive mapping of the repertoire of hand grasps available to human subjects, and are stored in the publicly available NinaPro Database

This section briefly presents the acquisition protocol, which was described in detail in the paper presenting the publicly available datasets [29]. During the experiment, the subjects were asked to sit at a desktop with the arms relaxed on the table and to repeat a set of movements with their right hand as naturally as possible. The entire experiment included up to 52 movements plus rest, divided into three exercises and extracted from the activities of daily living (ADL) and the hand taxonomy literature [20–23]. In this article, we consider only the set of hand grasps (Fig. 2) (i.e. the first 20 movements of the NinaPro exercise B [27]). The subjects were asked to repeat the movements represented in short films that were shown on the screen of a laptop with their right hand and they were asked to concentrate on mimicking the movements rather than on exerting high forces. Each movement was repeated 10 times for DB1 and 6 times for DB2 and DB5. Each repetition lasted 5 s and is separated by the other movements by 3 s of rest. The experiment was approved by the Ethics Commission of the Canton Valais (Switzerland). Before data acquisition, the subjects were given a thorough written and oral explanation of the experiment itself and were asked to sign an informed consent.

### Signal pre-processing

The data streams were over-sampled to the frequency of the fastest device (100 Hz for DB1, 2000 Hz for DB2 and 200 Hz for DB5) using linear interpolation. The movements performed by the subjects may not perfectly mirror the ones shown on screen due to human reaction times. Movement detection algorithms (i.e. the generalized likelihood ratio algorithm [27] and the Lidierth threshold based algorithm [30, 31]) were used to correct imperfect labeling.

### Calibration protocol and angle calculation

**Fig. 3:**
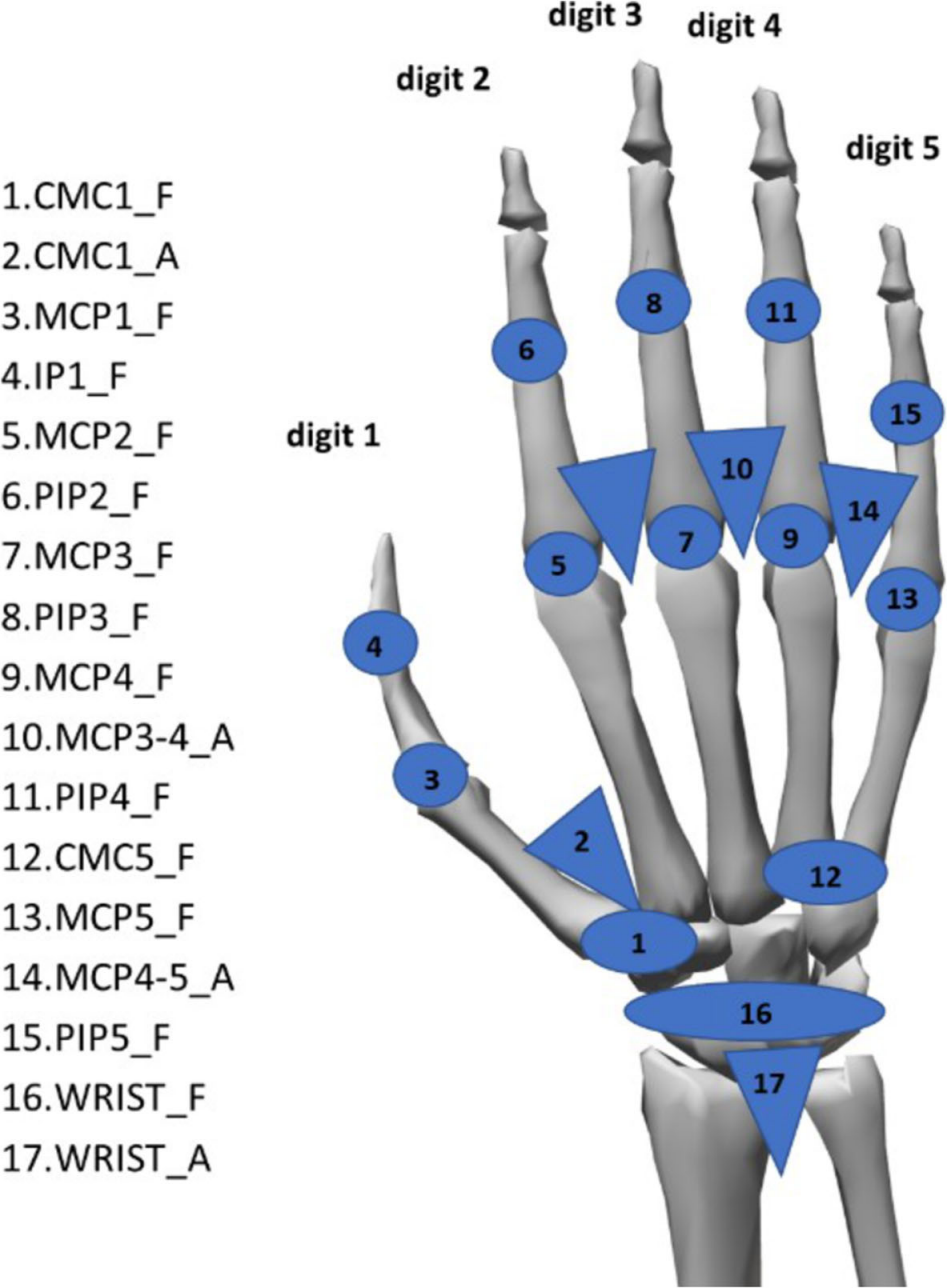
List of recorded anatomical angles. Nomenclature: _F for flexion (circles), _A for abduction (triangles); 1 to 5, digits

Joint angles were calculated from the raw data collected according to a precise calibration protocol [25]. The joints considered were: metacarpophalangeal flexion (MCP1_F to MCP5_F, 1 to 5 meaning thumb to little digits), interphalangeal flexion of the thumb (IP1_F), proximal interphalangeal flexion of the fingers (PIP2 to PIP5), flexion and abduction of the carpometacarpal joint of the thumb (CMC1), relative abduction between fingers MCPs (middle-ring and ring-little), palmar arching (CMC5), and wrist flexion and deviation (WRIST_F/A). The protocol was previously applied to 10 intact subjects and consists of registering 73 poses or guided movements to tune the gains of each sensor. This protocol includes the determination of gains and also corrections of cross-coupling effects for specific anatomical angles. The gains obtained through the calibration process were used to calculate the joint angles. More details about the correction procedures are available in the calibration protocol reference paper [25]. The corresponding 17 anatomical angles used in this study are shown in Fig. 3.

**Fig. 4:**
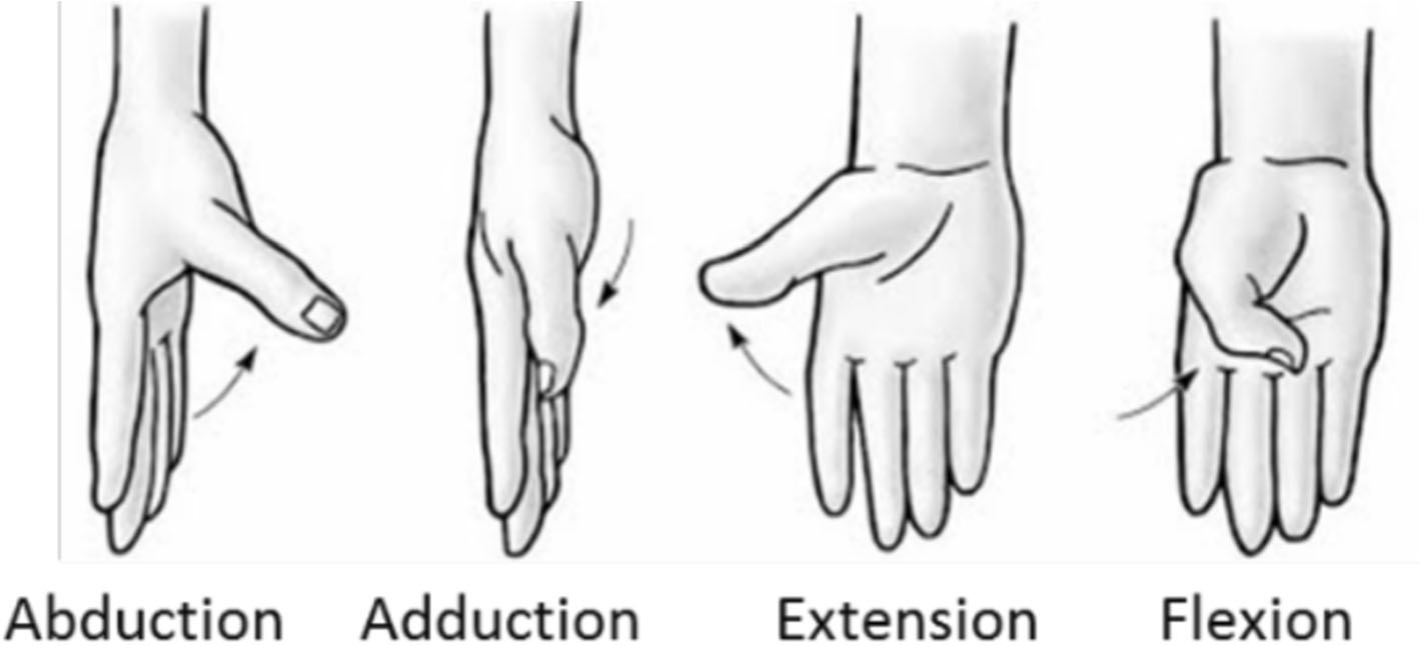
Sign criteria for the CMC joint of the thumb

Each joint excursion was assigned a sign (positive/negative) according to criteria shown in (Table 4 and Fig. 4).

**Table 4:** Sign criteria considered

PIP(2–5)_F, IP1_F, MCP(1–5)_F	Flexion + / Extension –
WRIST_F	Flexion + / Extension –
WRIST_A	Radial deviation + / Cubital deviation -
MCP(3–4, 4–5)_A	Fingers separated + / Fingers together -
CMC5_F	Flexion +/Extension -
CMC1_F	Flexion +/Extension – (See Fig. 4)
CMC1_A	Abduction +/Adduction - (See Fig. 4)

### Summary of the analysis steps

The following outcomes are presented according to these steps.

1. Kinematic PC extraction: Extraction of kinematic motor synergies of 20 grasps from each of the 77 subjects (using PCA, selecting PCs that have eigenvalues > 1). The following analyses are presented:
  definition of the dataset of synergies;
  number of extracted synergies per subject;
  amount of variance explained per subject.
2. Synergy clustering: Clustering of the whole dataset of extracted synergies (Hierarchical Clustering, cut off distance criteria when the highest distance is observed). The following analysis are presented:
  identification of the dendrogram for the extracted dataset;
  identification of the variance explained by each cluster;
  identification of the mean synergies;
  characterization of the mean synergies.

### Kinematic PC extraction

The Data Analysis was fully performed with MATLAB 2016a with custom-developed software. First, the re-labeled movement stimulus provided with the dataset was used to separate movement phases. Joint angles were filtered with a 2nd-order 2-way low-pass Butterworth filter with cut-off frequency of 5 Hz. In order to allow the comparison of all the acquisitions referring to each grasp, the number of frames of each movement repetition was rescaled to 1000, considering all phases (reaching, grasp and release) and all the grasps. Then, for each subject, the 17 joint angles of each rescaled movement repetition were pooled in aggregated matrices and synergies were extracted using PCA [32]. PCA was applied in order to extract the PCs, related to each subject’s motor behavior in hand grasps_:_ Each PC obtained is a vector that contains the loads (or weights) of each of the 17 original variables in a space where the original data were modified and grouped showing the coordination between them, in order to reduce the number of variables so that each of these new variables is no longer correlated with the other variables. For each subject, the PCs with eigenvalues higher than 1 were extracted (as one of the common criteria of component selection) with normalized factors and varimax rotation [33]. Finally, the number of the PCs extracted and the variance explained for each subject were presented.

### Synergy clustering

The extracted PCs of each subject were included into the cluster analysis. Conglomerate or hierarchical clustering analysis [32] is a multivariate technique that allows the classification of elements into groups or clusters, so that each element is very similar to those in its own conglomerate according to some specific selection criteria. In this case, as the pairwise distance between pairs of observations, the angles between PCs were calculated as in Eq. 1:

1 $$ \mathit{\operatorname{arccos}}\left(\left|\frac{PCL_i\ast {PCL}_j}{\left\Vert {PCL}_i\right\Vert \ast \left\Vert {PCL}_j\right\Vert}\right|\right) $$

i and j are ranging from 1 to N, where N is the total number of PCs extracted and PCL_ij_ are the vectors containing the PCs extracted. The resulting angles are arranged in a matrix of angles of size N x N. Then, this matrix was used as input to a hierarchical clustering procedure. As the algorithm for computing the distance between clusters, the “complete” algorithm was used. The PCs were hierarchically grouped depending on their similarity represented by their pairwise distance computed in Eq. 1. The desired number of clusters was identified by considering the distance between elements for each hierarchical step of the dendrogram tree. Considering all the possible couples of the following grouping steps, the elements were grouped when the highest distance between the clustered groups in a step was found (in comparison to the previous ones). Like this, the elements grouped at a specific level of the dendrogram are clustered at a higher hierarchical level, which may be more appropriate. Finally, the percentage of variance explained for each group was calculated as the number of PCs in each group divided by the total number of PCs. Therefore, considering the number of PCs grouped in each cluster, the first groups (or synergies) that explain at least the 80% of the total variance were represented and described physiologically.

## Results

### Extracted PCs

**Fig. 5:**
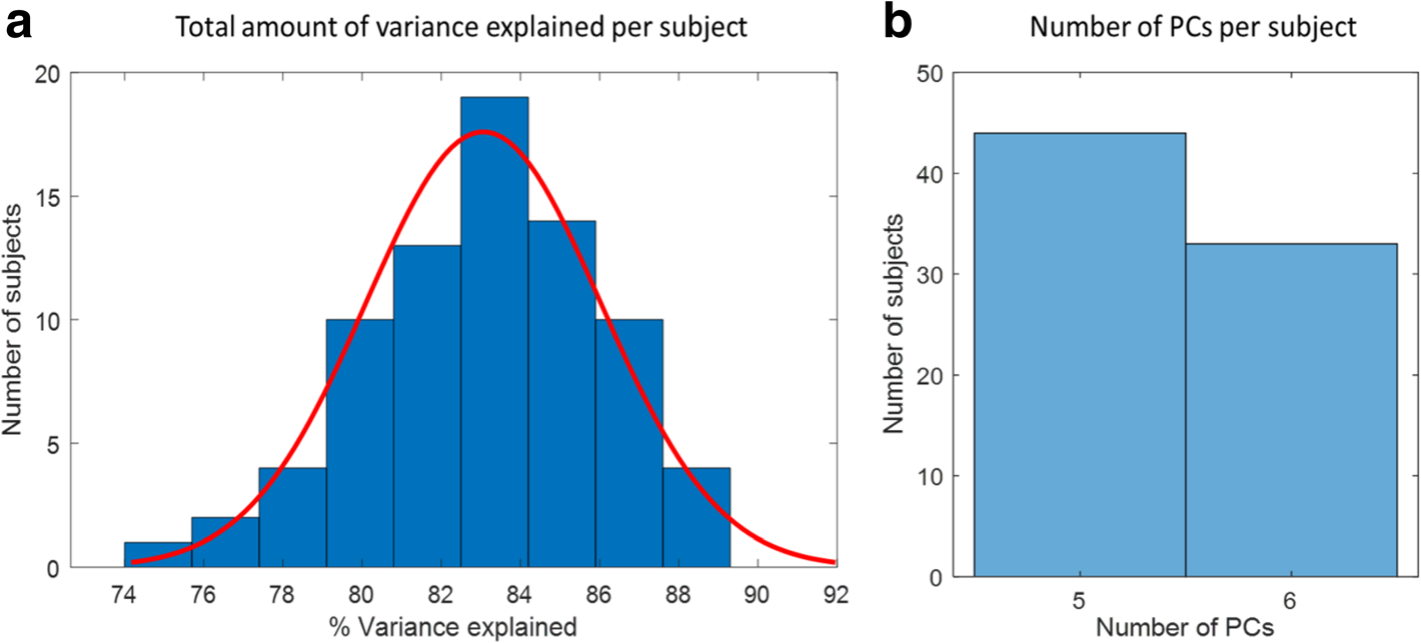
Histograms of % variance explained (**a**) and number of PCs (**b**) versus number of subjects

The number of extracted PCs as well as the total amount of variance explained per subject are summarized in Fig. 5 by portraying the subject distribution in a histogram. Five PCs were extracted in 44 subjects, and six PCs in 33 subjects. The histogram of variance explained for each subject, shows the peak of the data at about 82–84%. In addition, the data spread is from approximately 74 to 90%. The variance seems to fit a normal distribution. Therefore, the amount of explained variance for each subject, according to the criterion described in the methods paragraph, is 83.06 ± 2.96.

### Synergy clustering

**Fig. 6:**
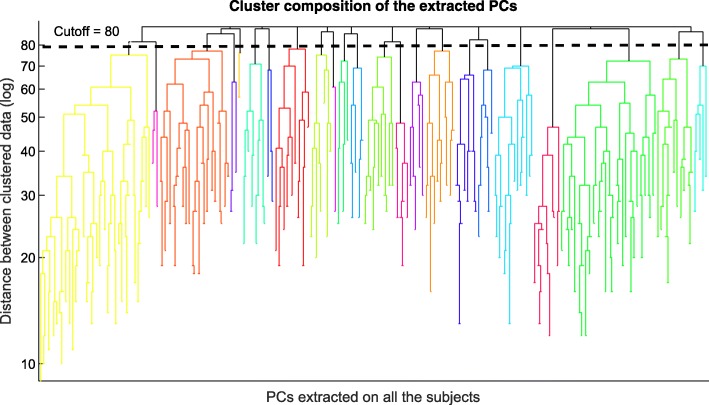
Dendrogram for cluster composition. Using angles between data as pairwise-distance and Complete linkage algorithms for cluster composition in the Dendrogram. The horizontal axis represents all the PCs with 418 numbers of clustered nodes; vertical axis represents the distance between the observed subjects in a logarithmic scale

The whole dataset of PCs that is composed of the 418 extracted PCs was clustered according to the hierarchical algorithm. Figure 6 shows the dendrogram used for the selection of the number of groups. The number of groups was chosen when the highest distance between the clustered groups in a step is found (in comparison to the previous ones). In this case, this highest distance corresponds to a cut-off of 80 pairwise distance, obtaining 23 groups of synergies.

**Fig. 7:**
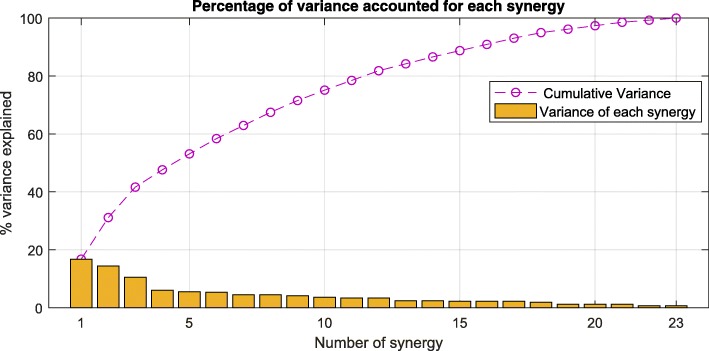
Percentage of the variance explained (calculated as the number of PCs in each group divided by the total amount of PCs) for each group of the 23 synergies found when cutting the dendrogram tree at a distance of 80

Figure 7 shows the graph with the metric used for the choice of a reasonable number of clusters as representation of the kinematic synergies of the dataset. We chose the number of synergies that explain at least 80% of this variance. Therefore, 12 synergies were considered a reasonable trade-off between accuracy and synthesis to be representative of all the subjects and grasps considered in this study.

**Fig. 8:**
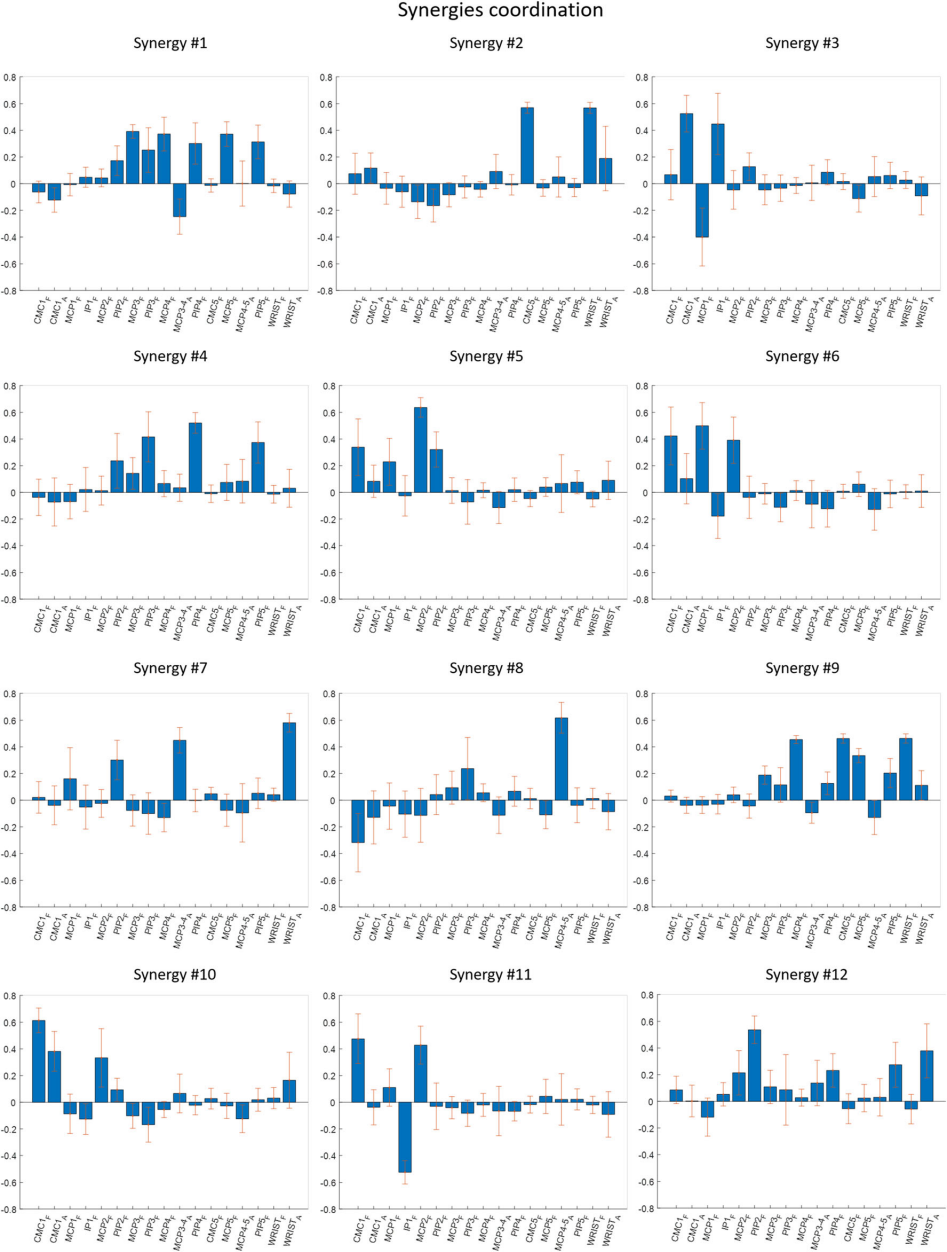
Bar and error plots representing mean and standard deviation of the loadings of different joints for the 12 synergies considered. The picture highlights the joint correlations represented by each synergy. Positive values represent flexion for PIP (2–5)_F, IP1_F, MCP (1–5)_F, WRIST_F, CMC5_F and CMC1_F; abduction for CMC1_A; radial deviation for WRIST_A; and fingers separated for MCP (3–4, 4–5)_A

The 12 grouped synergies are summarized in Fig. 8 by describing the mean and standard deviation of each synergy coordination between the joints of each synergy considered. A short description of the main aspects of each coordination pattern follows:

- Synergy 1: flexion of MCP of 3rd to 5th fingers, flexion of PIP of 2nd to 5th fingers and adduction of MCP of the 4th finger.
- Synergy 2: wrist flexion with palmar arch (CMC5) flexion.
- Synergy 3: a coordination movement of abduction of thumb CMC, extension of MCP and flexion of the IP of the thumb.
- Synergy 4: a PIP joint coordination.
- Synergy 5: a coordination between flexion of MCP and PIP joints of index finger, along with flexion of thumb CMC and MCP joints.
- Synergy 6: thumb CMC and MCP of the thumb, together with the MCP of the index finger.
- Synergy 7: wrist radial deviation with abduction of the MCP of the 4th finger. It also shows PIP flexion of the index finger.
- Synergy 8: an abduction/adduction movement of MCP of the 5th finger.
- Synergy 9: flexion MCP of 4th and 5th fingers, along with flexion of palmar arch (CMC5) and wrist.
- Synergy 10: flexion and abduction of CMC joint of the thumb along with flexion of MCP of the index finger.
- Synergy 11: flexion of CMC joint, extension of the IP of the thumb and flexion of MCP of the index finger.
- Synergy 12: flexion of PIP joint of the fingers with wrist radial deviation.

**Fig. 9:**
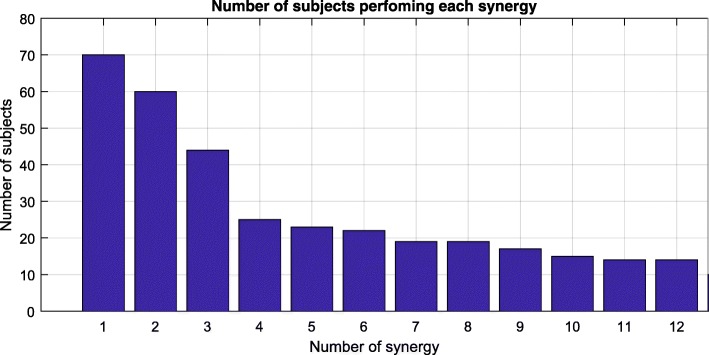
Number of PCs grouped in each of the 12 synergies selected (e.g. the first synergy was found in 70 subjects)

The number of PCs grouped in each of the 12 chosen synergies is also shown in Fig. 9. Synergies 1 to 3 are the most frequently repeated among the subjects. In contrast, Synergies from 4 to 12 are less prevalent and are only used by a small number of subjects.

### Comparison with related studies

Comparing the results with previous studies highlights that the number of synergies required to explain grasps variance is higher than what was previously described and that the first three synergies seem to explain sensibly lower variance. Both considerations can be related to the high number of subjects and movements. Santello et al. [4] recorded 15 joint angles in five subjects while performing imaginary grasping postures. In their work, two synergies explained up to 84% of the variance, corresponding to a lower dimensionality of the control space. The difference in the results can be due to several aspects, such as the exact setup, the number of subjects and the degrees of freedom. The similarity between the two approaches may be limited, as the work by Santello et al. did not mention any calibration procedure to obtain anatomical angles from raw data (which can be important to reduce problems related to non-linearities [24]).

The data standardization procedure followed in this study allowed us to compare joints with different range of motion, leading to a higher number of synergies having varying compositions. The number of DoF and of subjects also increased the number of PCs that are required to explain variance [10]. In Liu et al. [10], the authors found that 6 PCs were necessary to explain 80% of the total variance when 18 DoF were recorded. These results are consistent with our results, where 5 or 6 PCs explain in each subject 80% of the variance.

It was shown that healthy subjects use a reduced number of coordination patterns when performing a set of daily life grasps, and that some are very similar between subjects. The coordination of the three main groups (the fingers, the palmar arch, the wrist, and the opposition of the thumb) were found in almost all subjects, proving that this motor behaviour is consistent among subjects. Some of the synergies found are comparable to synergies found in the literature, although some of them are novel: The first synergy found is comparable to the first synergy described in previous studies [4, 9], even if it shows slight differences. The synergy shows the flexion of most fingers except the index. This result is novel compared with the synergies found in the literature and is in accordance with recent studies describing the independence of the index finger and of the thumb [34, 35]. The second synergy includes the coordination of palmar arch and wrist flexion. This synergy was not described in previous studies, since it includes joints that are not usually taken into account in dimensionality reduction. Our results on 77 subjects highlight that this synergy should be included in studies based on dimensionality reduction and used in rehabilitation, since it is common among several subjects. However, the interpretation of the physiological role of the abovementioned synergy should be addressed with care. In fact, since the position of the wrist is related to the spatial position and orientation of the object to grasp, this synergy is related to the grasping of objects located in front of the subject. It is necessary to study different spatial positions and orientations of the object with respect to the subject to promote the emergence of all the available coordination groups. The third synergy mainly represents the thumb joint coordination and it is also described in [11]. In the literature it generally explains little variation and therefore appears in higher order synergies. This result confirms the importance of the thumb when performing common grasps in activities of daily life and it supports the quantitative assessment that the thumb is the most independent finger [35]. Therefore, it suggests using this synergy in fields like rehabilitation and prosthetics. It is known that thumb joints and movements are the hardest ones to measure (and to calibrate) with the Cyberglove acquisition system. This result was obtained by the accurate calibration procedure and the transformation of the raw data into joint angles, which provided us with the precise measure of the thumb joint movements.

Only the first three synergies appeared in more than half of the subjects analysed: PIP flexion and MCP flexion and adduction of fingers 3 to 5 (synergy 1), palmar arching and wrist flexion (synergy 2) and thumb opposition (synergy 3). This suggests that the finer or more precise synergies are subject-dependent (such as the control of the index finger and its necessary coordination with the thumb). Therefore, each subject can use different strategies to achieve the goal, in accordance with what proposed by Patel et al. [8]. Specifically, synergies number 5, 6, 10 and 11 show varying coordination between thumb joints and index finger. Synergies number 7 and 8 show coordination between abduction/adduction of the fingers and wrist. Synergies number 4 and 12 show similar coordination of the PIP finger joints. Synergy number 9 shows the coordination of MCPs and palmar arch, being explainable as the coupling of the first and second synergy. The results indicate that coordination between wrist and hand joints is not obvious in grasping. While the coordination of the joints of the hand do not seem to be affected by the position of the object to be grasped, the coordination of the wrist is influenced [10]. In future studies, the hand and wrist synergies need to be considered for a separate analysis.

## Discussion

The objective of this paper is to provide a more general formalization of the motor modules underlying human grasps. The results are only partially in accordance with previous findings, that showed a considerable reduction in the dimensionality of the datasets [4, 10]. However, novel methods of the study and novel results contribute to clarify several limits of previous literature and foster the development of applications in rehabilitation and assistive robotics.

The novel methods of this study include: first, a high number of subjects (targeting the possibility to generalize the results); second, a set of grasps that well represent activities of daily living and rehabilitation needs (due to their type, number and completeness); third, a novel synergy extraction procedure; fourth, the use kinematic data calibration procedures; fifth, the use of standardization procedures (that allow a fairer comparison of joint movements); and, sixth, the use of a publicly available dataset (allowing other researchers to replicate or improve the study).

The number of subjects analyzed in this paper is over 7 times higher than previous studies, providing a more complete analysis of hand kinematics reduction and of the variability in subjects. In this work we analyze 77 subjects, while the number of subjects considered in previous papers was up to 10 subjects, limiting the possibility to generalize the results and not providing information regarding subject variability.

The grasps considered in this paper provide a better representation of hand grasps for rehabilitation and assistance needs due to their number, their relationship with daily living requirements and due to movement completeness. The study considers 20 hand grasps, while the number of grasps considered in previous work is in general lower (Table 1). Only two studies analyzed a number of grasps comparable to the one considered in this paper, but the first one considered imagined objects (rather than real ones) and only 5 subjects [4], while the second one measured a limited number of DoF and only 10 subjects [8]. The hand grasps included in this paper well represent rehabilitation and assistance needs since they were carefully selected according to activities of daily living and the hand taxonomy literature, in order to provide full autonomy in daily life [20–22]. Finally, the analysis performed in this study is done considering the entire movements, including reach and release phases that were usually not considered in previous studies despite being part of the coordination of daily life activity that is intimately related to hand grasps.

The method proposed in this study allowed obtaining individual synergies for each subject, differently from most of the previous studies that extracted synergies pooling together the data from all the subjects involved in the study. In this work, the synergies were extracted individually for each subject and then grouped by means of clustering: 418 PCs were extracted and clustered according to the hierarchical algorithm, obtaining 23 groups of synergies. This fact is useful to take into account subject-related variability.

Kinematic data were accurately calibrated before extracting synergies, thus improving their reliability. Many previous approaches extracted the synergies from the kinematic raw values obtained from kinematic measurement systems such as data gloves without the use of calibration. In this work the physiological angles of the hand were computed using a calibration method. Calibration is fundamental to have reliable data because nonlinearities and imperfect kinematic representations are normally expected by measurement systems such as data gloves [24, 25].

Standardized kinematic data was used in this work in order to ensure that the contributions from all the joints are equally weighted inside the extracted synergies. This procedure allows joints with lower range of motion to be adequately represented in the synergy extraction procedure. It made it possible to compare joints with different range of motion, leading to a higher number of synergies having different compositions.

All data used will be made publicly available on the NinaPro platform,^3^ in order to allow other groups validating and improving the results.

In terms of novel results, the number of synergies required to explain grasp variance is at least twice the number of synergies as other articles and the first three synergies seem to explain sensibly lower variance.

The PCA allowed to reduce the dimensionality of the dataset to a limited number of kinematic patterns underlying a large variety of hand movements and explaining the majority (over 80%) of the dataset variability. These results can be considered as a further experimental evidence of the modular organization of the control strategy of the central nervous system. However, the dimensionality of the control space found in this study is considerably higher than found in previous studies: 12 kinematic synergies explain 80% of the total variance, which is more than twice the number of synergies described in the literature. This has several reasons: the number of DoF considered, the large number of subjects, the individual extraction of synergies, the standardization and calibration methods used in the kinematic data before the PCA as well as the high variability of grasps performed (considering also the whole movement).

Another novelty is that less variance is explained by the three main synergies (~ 40%) than in the other studies (Table 2). This seems to suggest that considering a large number of subjects as well as studying complex grasping movements may actually modify the number and type of synergies.

The paper also contains several limitations. First, even if the best and to our knowledge most accurate methodologies were used, signal pre-processing can be a source for alteration of the extracted synergies. Second, synergy extraction procedures have inherently limitations and can be influenced by the choice of the extraction method, differences in movement replication by the subjects, grasp choice etc. While increasing the number of subjects and the number of grasps can limit the practical effect of movement and subject variability, deeper analyses needs to be performed to analyze the pre-processing and the extraction procedure effects.

The kinematic and muscle synergies were rarely coupled. A recent study mapped the muscle synergies during hand grasps on data of the NinaPro database obtaining a similar number of modules [36]. This result suggests that the modular organization is employed at both muscle and kinematic level.

The use of the modular organization of the neuro-motor system, expressed in terms of muscle synergies, is a long-established concept in rehabilitation. Changes in the number, structure and recruitment of motor patterns may allow to discriminate a variety of pathological changes in the nervous system [37]. Kinematic parameters describing movement capabilities are mostly reported for chronic patients and correlate strongly with clinical assessments. However, further studies on measures to assess coordinated movement have been recommended [38]. In this perspective, the results obtained in this study can reveal biomechanical constraints and motor control strategies employed by healthy populations. As a consequence, the identified 12 synergies can characterize the most representative coordinated joint movements in order to define kinematic benchmark patterns to study how neurological injuries alter motion coordination patterns and cause impairment.

Many devices are already used for the rehabilitation of the hand and to restore the motor functionality of the hand and fingers [39], showing that improvements can be achieved by monitoring and training specific physiological parameters [40]. Synergy-based paradigms might allow promoting motor re-learning by focusing on the patterns that are implemented at the neural level. Recently, gloves for rehabilitation have been designed according to the concept of motor primitives [41] in order to provide synergy-based recognition of grasps. Recent reviews highlight that hand-rehabilitation devices are available in many applications, since they are fundamental to restore full interaction with the environment [42]. A better understanding of kinematic synergies as well as of muscular synergies [36] may allow to improve the knowledge on grasp patterns and to associate them to actuated gloves for rehabilitation.

The described results on kinematic hand synergies can lead to applications in prosthetics and possibly in industrial manipulation. Robotic hands that reproduce hand movements by modulating the main postural hand synergies were recently presented [13, 43]. However, improving them with models that generalize to many subjects and to comprehensive grasps for activities of daily living (including reach and release phases) can strongly improve these prostheses. The improved models can provide the prostheses with higher functional adaptability, making them more compatible with different subjects. Models that are computed on grasps targeting activities of daily living can lead to prostheses that better interact with the environment in real life conditions. Including reach-to-grasp and release phases into the models can as well improve the prostheses, making them more similar to real hands and possibly empowered with multis-sensorial inputs (e.g. [44–46]). Finally, if the results are matched with muscular correspondents, they may lead to real natural and continuous myoelectric control of robotic hands, a challenge not yet achieved to the best of our knowledge.

Thus, the impact of this paper is expected to be in a more accurate and comprehensive mapping of the human kinematic synergies to be employed for evaluation and control logics of devices, according to all the novel elements described. The findings will provide possibilities to implement more realistic and complete training and evaluation paradigms that take into account the particularities of human motor control.

## Conclusions

In this study, a reference set of hand kinematic synergies is provided. The synergies were extracted from the largest database of kinematic hand grasps currently available that will also be publicly available. The data include 77 subjects performing 20 hand grasps, each repeated 6 times. Moreover, the analyzed data are calibrated in angles (thus taking into account non-linearity related to the Cyberglove), they use standardization procedures and allow the confirmation and improvement of the study (thanks to the public availability). Thanks to the high number of subjects, the characteristics of the considered grasps and the pre-processing procedures, the results currently represent he most complete and precise set of kinematic hand synergies related to activities of daily living.

Several of the synergies described in previous studies are confirmed. In particular, the flexion and adduction of the MCP of fingers 3 to 5 (synergy 1), palmar arching and flexion of the wrist (synergy 2) and opposition of the thumb (synergy 3).

However, the results show several differences from previous studies. The number of synergies required to explain grasp variance in our study is at least twice the number of synergies of other articles and the first three synergies seem to explain sensibly lower variance. Minor synergies show higher variability, probably also due to the high number of subjects and sensors [8]. Finally, the results highlight the importance and independence of the thumb and of the index.

Future work is planned to involve the use of this set as a tool to evaluate motor repertoire of impaired people and design accurate rehabilitation paradigms to train desired coordination patterns.

## Abbreviations

ADL: Activities of daily living
CMC: Carpometacarpal
DoF: Degrees of freedom
IP: Interphalangeal
MCP: Metacarpophalangeal
PCA: Principal Component Analysis
PCs: Principal components
PIP: Proximal interphalangeal

## Footnotes

1
http://ninaweb.hevs.ch/

2
http://zenodo.org/

3
http://ninapro.hevs.ch/
